# Peer Review of “Assessment of SARC-F Sensitivity for Probable Sarcopenia Among Community-Dwelling Older Adults: Cross-Sectional Questionnaire Study”

**DOI:** 10.2196/77582

**Published:** 2025-07-25

**Authors:** Anonymous

**Keywords:** sarcopenia, neuromuscular, screening, community, scale, measure, questionnaires, diagnosis, gerontology, older adults, muscular

*This is a peer-review report for “Assessment of SARC-F Sensitivity for Probable Sarcopenia Among Community-Dwelling Older Adults: Cross-Sectional Questionnaire Study.”*

## Round 1 Review

### General Comments

This paper [1] conducted a validation to derive a cutoff value that predicted low grip strength from SARC-F (strength, assistance with walking, rising from a chair, climbing stairs, and falls) scores and showed that the cutoff for SARC-F scores is 2 points. Many issues need to be resolved before this study can be published.

### Specific Comments

#### Major Comments

1. The study looked at the association between SARC-F and grip strength, which is not novel. Sarcopenia is poorly defined.
2. The sample size needed to be more adequate, and only 11% of the subjects had lower grip strength.
3. It is acceptable if it is used for estimation or prediction, such as death, but an area under the curve of 0.77 may be too low as an index for diagnosis and discrimination.
4. The Methods describe too few details, and Table 1 provides too little background information.
5. Ultimately, the conclusions that can be drawn from the results should be revised.

## Round 2 Review

### General Comments

The authors have attempted to revise the manuscript to the best of their ability, but even so, this study seems to lack important points.

### Specific Comments

#### Major Comments

To begin with, SARC-F is a screening indicator for sarcopenia, not for probable sarcopenia (decreased grip strength). If you try to find a cutoff for probable sarcopenia, which is a prestage of sarcopenia, the cutoff value will inevitably be smaller than the cutoff value used to determine sarcopenia. With that in mind, how do you explain the significance of this paper? Please argue the need to screen for decreased grip strength with a cutoff of 2 points rather than screening for sarcopenia with a cutoff of 4 points.

In addition, the cutoff of 2 points on a questionnaire consisting of five items with a range of 0‐12 points is an extremely low value. The question that arises here is whether there is any point in using this questionnaire in the first place. The authors will first need to show which of the lower-level items contribute strongly to the prediction of grip strength decline as a sensitivity analysis. Then, they should also mention whether the SARC-F should be used as a questionnaire indicator or whether it would be better to use the lower-level items as a new screening indicator.

#### Minor Comments

Information on ethical matters is lacking.

1. Is there an ethics approval number?
2. It is said that informed consent was not required, but how was information disclosed to the research subjects regarding your research? Was an opt-out notice posted?
3. How was the opportunity for the subjects to decline participation in your research provided?

It says “regularly scheduled physician visits,” but is this study a single or multicenter study?

What is the reason for the subjects’ physician visits? Are the subjects suffering from some disease? If so, the disease information may be an important confounding factor in this study, so please clearly state the results and show them in Table 1.

Please show the inclusion and exclusion criteria for the subjects.

Who measured grip strength, where, and in what position?

In the Statistical Analysis section, it says “visual histograms,” but they are not shown in the Results. Please show them. In particular, it would be desirable for the histogram of the SARC-F score to be free from extreme bias when conducting the analysis. Please show the histogram for each sex and show that the sampling is appropriate for verifying the value conducted in this study.

Before validating the cutoff value of the SARC-F based on grip strength, it’s crucial to establish a robust relationship between grip strength and the SARC-F. This can be achieved through multiple regression analysis, with grip strength as the dependent variable, the SARC-F as the explanatory variable, and other factors as adjustment factors. This step is essential to ensure the validity of the research.

The factors that may confound the relationship between SARC-F and grip strength have yet to be sufficiently demonstrated. For example, what about cognitive function and physical activity?

The male’s grip strength of 36.3 kg is extremely strong for a subject who should be selected for probable sarcopenia. There is a high possibility of selection bias. Please clearly state in the Discussion how you interpret this point.

As mentioned above, much important information needs to be included, and even though there are limitations from the research planning stage, they should be mentioned in the Discussion.

If you do not present the information mentioned above, please clearly state the limitations of the research in the Discussion section, and also explain why you still think the research results are meaningful and why it is necessary to make the results of this research public.

## Abbreviations

SARC-F: strength, assistance with walking, rising from a chair, climbing stairs, and falls
